# The Present-Day Mortality of Pneumonia

**Published:** 1902-12

**Authors:** Alex G. Brown

**Affiliations:** Richmond, Va., Adjunct Professor Practice of Medicine, University College of Medicine


### THE PRESENT-DAY MORTALITY OF PNEUMONIA.*

BY ALEX G. BROWN, JR., RICHMOND, VA.,
Adjunct Professor Practice of Medicine, University College of Medicine.

In the ancient history of disease no subject has received more careful description and study than has pneumonia. The physicians of antiquity, of Grecian, Roman and Arabian nationality, set forth their knowledge of the pneumonia of that day in descriptions filled with many interesting conceits and queer observations. In the writings attributed to Hippocrates and Aretæus, among the symptoms mentioned, we find these: "the tip of the nose is turned up;" "the white of the eye has a greasy lustre;" and others as curious, while "the diagnosis is made by the coating of the tongue."

For many decades greatest confusion reigned with reference to pneumonia, in fact pleurisy and pneumonia really were not differentiated until 1819, when Laennec pointed out these distinct and separate diseases; to him is due the classification of the stages of pneumonia which at the present time is accepted as correct. However, through all the writings of history, this fact is constantly set forth, though statistics are not given that pneumonia is a deadly disease.

In casting about for a subject for discussion on this occasion, I have settled on "The Present-day Mortality of Pneumonia and its Prophylaxis" as a proper one. So great as a factor in the death-rate has pneumonia become, and so rapid and terrible its brief course, that I have deemed its consideration not an unprofitable pursuit. At the outset, I wish to comment upon two facts which have a direct bearing on this question of pneumonia mortality; first, the advances of science have, during the last decade, raised the average life in the United States 4.1 years; in the decade ending at 1890 the average life-length was 31.1 years, while in the decade ending 1900 the average life-length was 35.2 years—a marked and gratifying increase. Second, the "great white plague" or tuberculosis, which up to recent date has exacted, of all dis-

*Read before the Richmond Academy of Medicine and Surgery, November 11, 1902.

eases, the greatest toll on human life, even to one-seventh of the world's mortality, is considered now to be on a decline. This may be explained by the world-wide fight which has been made recently against its progress. For the universal interest in civil, social and scientific circles, as manifested in international conferences of scientific men, in popular education of the masses, and in state, municipal and private grants of large sums of money for the study of prophylaxis, and treatment of this erstwhile captain of death, has begun to have a most gratifying effect upon the mortuary table.

Now, pneumonia, as a swift destroyer, has appeared, according to latest statistics, proving itself to be man's most formidable enemy, yielding to no other in the number of its deaths, and certainly effecting this end with swiftest brevity. Indeed, may not this ancient disease treated through the ages, with only this grave statistical result, justly claim the sober consideration now accorded it by the profession?

According to the vital statistical report of the registered area of the United States, in the twelfth census bulletin of August 20, 1901, pneumonia holds leading place in the death column, having been the cause of death in 1900, in 55,296 instances, i. e., 191.9 persons in 100,000 population. This fact is borne out by various particular localities which I will cite to establish well this point. In the report of the department of health of Chicago, we find that between 1851-1890, there were 25,719 deaths from consumption and 16,577 deaths from pneumonia; i. e., more than 35 per cent. excess of consumption deaths. Now, between 1891 and 1901 there were 22,957 deaths from consumption and 25,228 from pneumonia, making an excess during this last decade of 9 per cent. of the latter. From the same report we find in the decade 1861-1871, deaths from pneumonia formed 3 per cent. of all the total deaths from all causes; in the two succeeding decades they were respectively 5 per cent. and 6.7 per cent. of the total deaths. And the last decade, 1891-1901, the proportion rose to more than 10 per cent. (10.2) of the total deaths from all causes.

In Massachusetts, also, we find a great increase in death-rate from pneumonia, it having risen from 7 to 8 per cent. in the late

'70's, to 9 and 10 per cent. in the last decade. Also, in Rhode Island and Connecticut, we find a similar increase of pneumonia death-rate and, doubtless, as says the editor of the *Journal of the American Medical Association*, were the figures available we would probably find a corresponding increase throughout the northern Atlantic and lake States. In Virginia we find for the year ending May 31, 1900, there were 25,252 deaths from all causes; of this number 2,429 died from pneumonia, i. e., about 10 per cent. In Richmond, Va., we find there were 2,523 deaths from all causes during the year ending May 31, 1900, and of that number 239 were deaths from pneumonia, or 9.4 per cent.

In order to explain this state of the mortality table, let us briefly set forth some of the influences which seem to tend to aid the *occurrence* of pneumonia. One of the chiefest factors operating as a recent causal agent may be the great fourth pandemic of influenza. It began, as Osler says, October, 1889, in some of the distant provinces of Russia, and by the following November Berlin was attacked. By the middle of December it was in London, and by the end of the month it had invaded New York, and was rapidly distributed over the entire continent. Accordingly, in the following year, pneumonia death-rate in Chicago doubled its former rate, and throughout the entire country there was a marked increase in its death-rate. Another factor in this increase of pneumonia deaths may be the lessened death-rate of the newborn and infants from intestinal disease. Many infants kept alive in delicate health, may readily succumb before childhood is over to pneumonia. At the other end of life the years have been increased and a greater number of aged lungs, in the sudden change of weather, and in exposure to atmospheric and microbic dangers, may become ready victims of pneumonia. The common use of alcohol to excess, the rheumatic and gouty diathesis of modern day life, the concentration and overcrowding of city living, the dusty and filth laden air of the city street way, all may tend to increase the rate of death from pneumonia.

In any discussion of the present-day mortality of pneumonia, to omit to mention its communicability and discuss its prophylaxis would be to omit a most essential part. According to the latest authorities, pneumonia is classified as an infectious disease, due to the germ pneumococcus of Fraenkel, in at least 95 per cent. of cases, the remaining 5 per cent. being accredited to other germs, namely, streptococci, Pfeiffer bacilli, pneumococcus of Friedlander. This germ is non-motile, occurs in pairs, oval or lancet-shaped, surrounded by a substance like mucin. It grows in alkaline culture media, with or without oxygen, at 35° to 38° C. It is self-limited of life in any media in four to five days, possessing, however, in the dry state great latent virulence for a long time, especially when fostered in desiccated sputum.

Now, to establish the communicability of this germ let us cite a few instances of notable epidemics. Tyson cites the case of a ship's crew of 815, of which 410 were attacked by pneumonia in rapid succession, and before the epidemic had subsided 720 had the disease, the fearful number of 298 having perished. Dr. Cunningham, of Alabama, reported at the last meeting of the American Medical Association, at Saratoga, a series of epidemics that had occurred during the last fifteen years, among the prisoners under his charge. In 1886 Darlington treated a hundred and five cases among laborers living in adjoining huts. In 1888, in Middleborough, England, there occurred 367 cases of pneumonia in a population of 40,000. The history of epidemics in foreign prisons, garrisons and armies, and in native asylums, institutions and boarding-schools is too extensive; suffice it to say that pneumonia is an infectious and communicable disease, modified and influenced by such predisposing factors as age, sex, race, former attacks, unsanitary living, exposure to cold, occupation, rheumatic diathesis, anesthetization by chloroform and ether and surgical operations.

Passing through the history of the treatment of pneumonia by blood-letting, by tartar emetic, by blistering, by veratrum viride, by calomel in large doses, by elimination of the toxins, by use of oxygen and care of the heart with diffusible stimulants and antipyretics, we take up that neglected phase of the question, prophylaxis.

Dr. N. S. Davis, Jr., in a recent article, makes this pertinent inquiry in discussing this subject: "But is the medical profession altogether free from blame for its (pneumonia) prevalence?" and later he says "prophylactic measures have not been enforced a they should have been." The public education and professiona tutorage of the masses, followed in the masterly fight of these latter years against the increase of tuberculosis, may be well repeated in the fight that should be made against the further increase of pneumonia death-rate. As in consumption, it must be not so much in the remedial measures as in the prophylactic agencies that we shall find the greatest results.

In the sick-room the physician must intelligently teach its dangers and its preventive measures to the attendants. In the training school for nurses he must expound the truth of its deadly communicability and its successful prevention. He must advocate such laws of public sanitation in regard to location, number, ventilation, disinfection of dwellings as will best prevent the spread of this germal disease. It should be the especial care of the profession to guard the aged and protect the infants from becoming victims of the prevailing evil conditions that so quickly cause them to succumb to pneumonia. With professional forethought in the treatment of influenza to avoid any complication of the lung, wise precautions in the administration of general anesthetics, the performance of surgical operations and the most careful sanitary and aseptic measures in the treatment of pneumonia cases, much will have been done toward lessening the mortality.

In this day of medical enlightenment and the retrograde of fatality and frequency of all other infectious diseases, a masterful inactivity in the prophylaxis of pneumonia will but take from our day and time the glory of its wonderful progress as well as prove a deadly monster in the life of the human family; while if these seemingly overexacting measures of prevention be adopted, much will be done to annihilate the germal cause, check the fatal progress and protect the stronghold of all future good health against the devastating inroads of pneumonia.

BIBLIOGRAPHY.

Cyclopedia of Practice of Medicine—Ziemssen.
Twentieth Century Practice of Medicine—Vol. XVI.
Tyson—Practice of Medicine.

Osler—Practice of Medicine.
*Journal of the American Medical Association* (several copies).
*Census Bulletin*, August 20, 1901.
*Monthly Bulletin*, Department of Health, Chicago.
*New York Medical Journal.*
*American Medicine.*
No. 416 West Grace street.

